# PW03-006 - IL-1-B inhibition in Schnitzler’s syndrome

**DOI:** 10.1186/1546-0096-11-S1-A232

**Published:** 2013-11-08

**Authors:** HD De Koning, J Schalkwijk, J Jongekrijg, M Stoffels, JW van der Meer, A Simon

**Affiliations:** 1 Dermatology, Radboud University Nijmegen Medical Centre y, Nijmegen, Netherlands; 2 Radboud University Nijmegen Medical Centre, Nijmegen, Netherlands; 3 Internal Medicine, Radboud University Nijmegen Medical Centre, Nijmegen, Netherlands

## Introduction

Schnitzler’s syndrome is a chronic disabling autoinflammatory disorder, characterised by chronic urticaria, paraproteinemia and systemic inflammation. The interleukin (IL) 1 receptor antagonist anakinra is a very effective treatment, but requires daily injection and blocks both IL-1α and IL-1β. Canakinumab is a selective human monoclonal anti-IL-1β antibody with a long half-life.

## Objectives

We investigated the long-term efficacy and safety of canakinumab in Schnitzler’s syndrome.

## Methods

In an open-label, single-treatment arm trial, eight patients with Schnitzler’s syndrome received monthly injections with 150 mg canakinumab subcutaneously for 6 months, followed by a 3-month observation period. Primary outcome was complete or clinical remission at day 14. Secondary outcome measures included inflammatory markers, quality of life, time to relapse, safety and tolerability.

## Results

After stopping anakinra, patients developed moderate to severe clinical symptoms. Canakinumab induced complete or clinical remission at day 14 in all eight patients. Median C-reactive protein concentrations decreased from 169 mg/l at baseline to less than 10 mg/l on day 14 and remained low or undetectable. One patient discontinued participation on day 39 because of return of symptoms while all others remained in complete or clinical remission during the 6-month treatment period. Relapse after last canakinumab dose occurred within 3 months in four patients. For two patients, remission continued several months post-study. Five patients reported at least one adverse event, predominantly mild upper respiratory tract infections. One patient died in a traffic accident.

## Conclusion

In this 9-month study, monthly 150 mg canakinumab injection was an effective and well-tolerated treatment for Schnitzler’s syndrome. Our data demonstrate that IL-1β plays a pivotal role in this disease.

## Disclosure of interest

None declared

